# Supplementary material for: Genome-enabled discovery of evolutionary divergence in brains and behavior
Source: Sci Rep. 2021 Jun 21;11:13016. doi: 10.1038/s41598-021-92385-8 (PMC8217251; doi:10.1038/s41598-021-92385-8)
Supplement: Supplementary file 1 — Supplementary Legends. [file 41598_2021_92385_MOESM1_ESM.docx]

Contents

[Supplementary tables: 1](#_Toc72130089)

[Supplementary Table 1: Species and sequence characteristics 1](#_Toc72130090)

[Supplementary Table 2: Genome-wide identity of enrichment with topgene, SFARI and enhancer categories 1](#_Toc72130091)

[Supplementary Table 3: RNA-seq differential expression 2](#_Toc72130092)

[Supplementary Table 4: Overlap of Rock-Sand and Pit-Castle differentiated genes 2](#_Toc72130093)

[Supplementary Table 5: Samples for RNA-seq 2](#_Toc72130094)

[Supplementary figures 3](#_Toc72130095)

[Supplementary Figure 1 3](#_Toc72130096)

[Supplementary Figure 2 4](#_Toc72130097)

[Supplementary Figure 3 6](#_Toc72130098)

[Supplementary Figure 4 7](#_Toc72130099)

[Supplementary Figure 5 8](#_Toc72130100)

[Supplementary Figure 6 9](#_Toc72130101)

# Supplementary tables:

## Supplementary Table 1: Species and sequence characteristics

This table contains a list of the Species used for genome sequencing. We describe the source of the tissue, Accession number for the Genome, along with mapping percentage and mean coverage for the current Reference Genome assembly (UMD2a) and the previously used Reference Genome assembly (UMD1).

## Supplementary Table 2: Genome-wide identity of enrichment with topgene, SFARI and enhancer categories

This table contains rock-sand differentiated genes identified by the genome scan.

Tab1 is the cichlid gene list.

Tab2 are the genes also found in the SFARI dataset

Tab3 are the genes also implicated in the Neurocristopathy dataset

Tab4 are the genes that have a fixed SNP in a known craniofacial element enhancer element

Tab5 are the genes that have a fixed SNP in a known HCNC enhancer element

Tab6 is the gene set from Tab1 converted to Human analogs for enrichment testing.

Subsequent tabs are enrichment categories from the TOPFUN enrichment analysis

## Supplementary Table 3: RNA-seq differential expression

This table contains genes differentially expressed between socially rock and socially sand F_1_ brains.

Tab1 is the cichlid gene list.

Tab2 is the gene set from Tab1 converted to Human analogs for enrichment testing.

Subsequent tabs are enrichment categories from the TOPFUN enrichment analysis

## Supplementary Table 4: Overlap of Rock-Sand and Pit-Castle differentiated genes

This table contains genes that overlap between genes from Supplementary Table 1 (rock versus sand divergent genes) and the data described in Supplementary Figure 6 (pit versus castle divergent genes). Tab1 contains the identity of overlapping genes.

Subsequent tabs are enrichment categories from the TOPFUN enrichment analysis

## Supplementary Table 5: Samples for RNA-seq

This table describes the F_1_ hybrid crosses used for the brain transcriptome sequencing.

# Supplementary figures

## Supplementary Figure 1


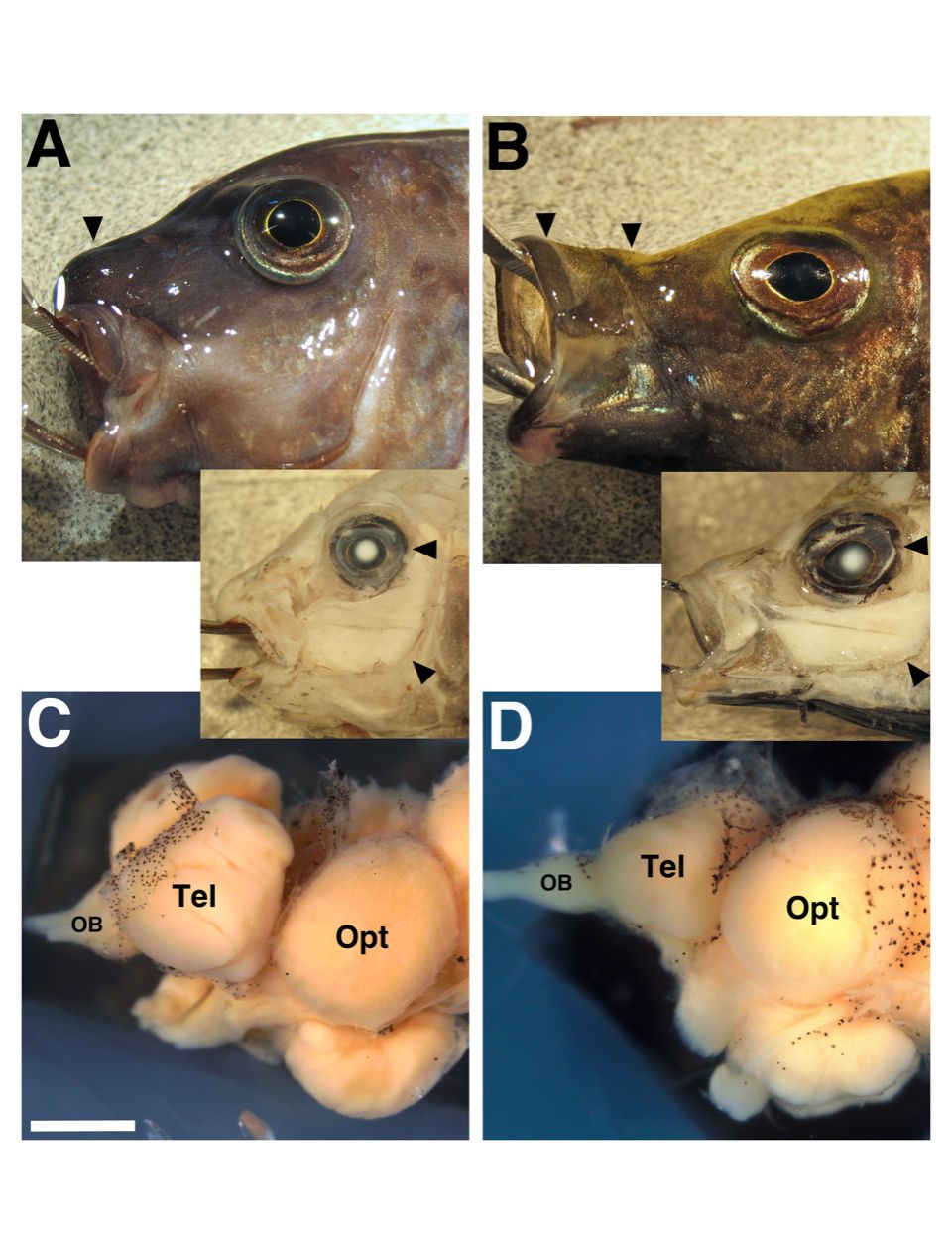


Supplementary Figure 1: Differences in the face (A-B) and brain (C-D) between rock- vs. sand-dwelling Lake Malawi cichlids | Rock-dwellers (A, C) have strongly reinforced jaws, smaller eyes, typically larger cheek muscles, larger olfactory bulbs and telencephala. Sand-dwellers have kinematic, gracile jaws, larger eyes, less robust cheek musculature, and large optic tecta.

## Supplementary Figure 2


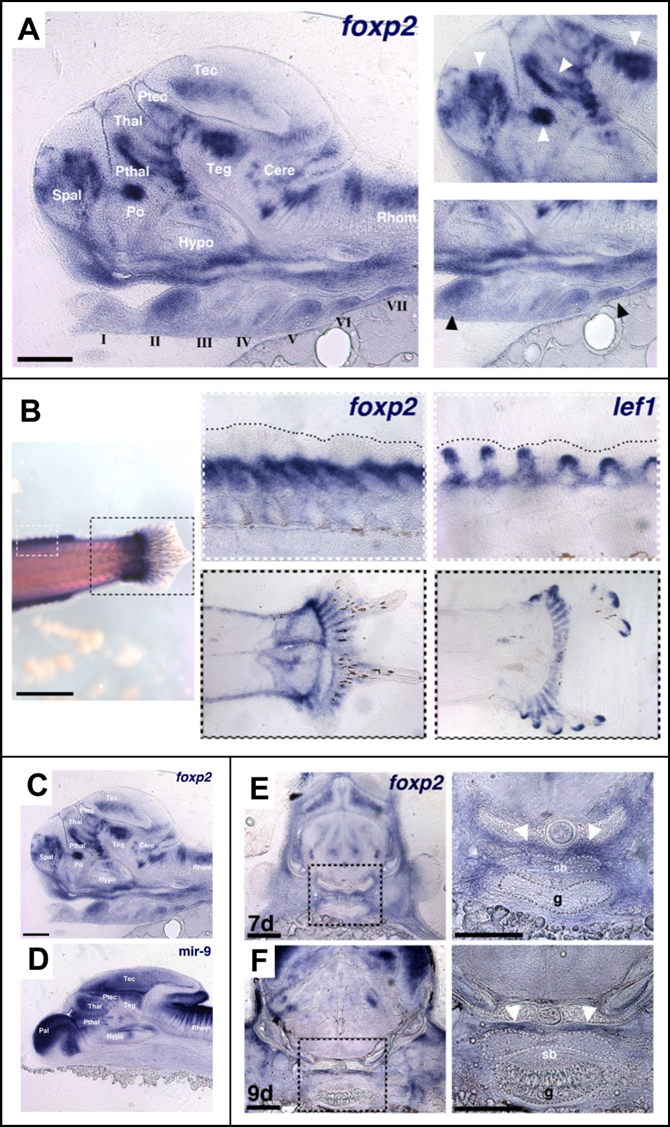


Supplementary Figure 2: Malawi cichlid *foxp2* is expressed in the brain and in all sonic organs | We previously noted novel expression domains for cichlid *foxp2* ^1^, elaborated here (A) Expression of *foxp2* throughout the brain (regions labeled in white) and developing pharyngeal arches (labeled I – VII) at 5 days post fertilization (dpf). On the far right, white arrows indicate expression in developing ganglia in the midbrain, diencephalon, pre-optic region, and subpallium. Black arrows point to expression in pharyngeal arches II and V. (B) *foxp2* expression, along with the WNT pathway transcription factor *lef1*, in the developing dorsal and tail fins at 7 dpf. The white- and black-dashed boxes on the right-most panels are zoomed in, midline sections of the boxes on the left. Adjacent, overlapping expression of *foxp2* and *lef1* are indicative of interaction between *foxp2* and the WNT pathway ^2^. (C) and (D) show the expression of *foxp2* and *micro RNA-9* (*mir-9*). *mir-9* has been shown to regulate *foxp2* activity in vertebrates ^3^ and the anti-correlated expression patterns in cichlids suggest a similar interaction. (E) and (F) document expression of *foxp2* in the developing swim bladder at 7 and 9 dpf. The black-dashed boxed on the left panels indicate the zoomed panels on the right. *foxp2* is generally expressed in the mesenchyme within and dorsal to the swim bladder at 7 dpf (white arrows). Once the swim bladder epithelium forms by 9 dpf, *foxp2* expression is localized dorsally (white arrows). All scale bars are 100μm. Abbreviations: Rhom = Rhombencephalon, Cere = Cerebellum, Teg = Tegmentum, Tec = Optic Tectum, Ptec = Pretectum, Thal = Thalamus, Pthal = Prethalamus, Po = Pre-optic area, Hypo = Hypothalamus, Spal = Subpallium, sb = swim bladder, g = gut.

## Supplementary Figure 3


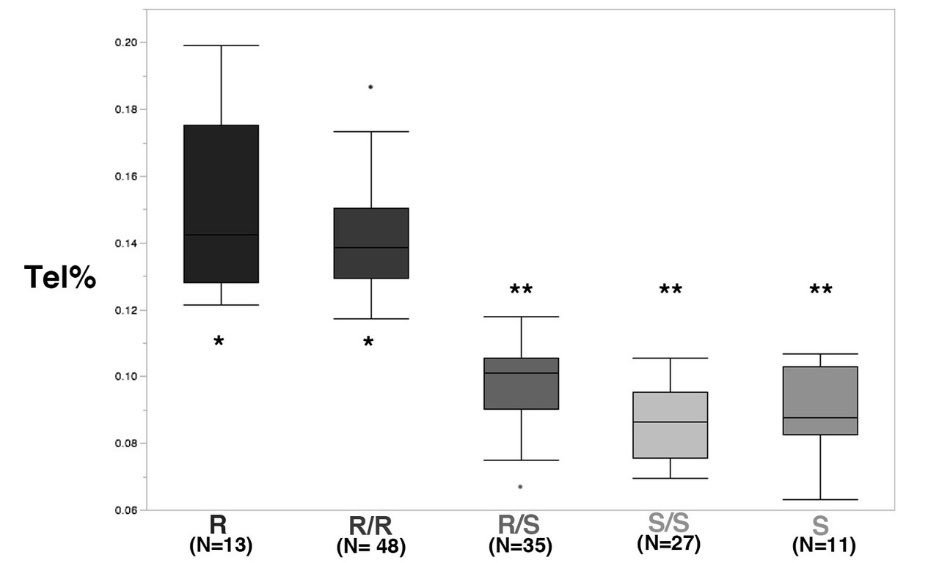


Supplementary Figure 3: The relative size of the telencephalon differs in rock-, sand- and F_2_ hybrids indexed for *irx1b* genotypes | Rock-, sand- and F_2_ hybrid individuals indexed for *irx1b* genotype were sampled at stages 12-14. We calculated the volume of the telencephalon in each individual and express this as a percentage of total forebrain volume. The rock individuals and R/R homozygotes (*) were statistically different than R/S heterozygotes, S/S homozygotes and sand individuals (**; Tukey’s test, p<0.0001). See also Figure 2.

## Supplementary Figure 4


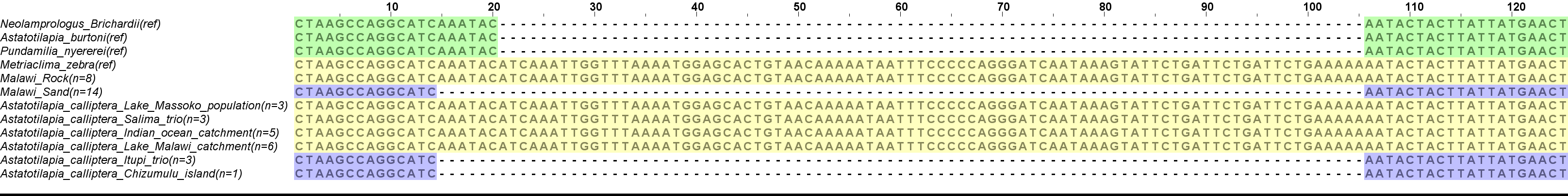


Supplementary Figure 4: Schematic of an InDel located in the 3’UTR of the Malawi cichlid *irx1b* gene drawn using Jalview 2^4^. Summary of allelic states: (1) rock-dwelling species possess an 85bp insertion (yellow) with similarity to Rex1 non-LTR retrotransposon, compared to outgroup species (shown in green); (2) sand-dwellers generally lack the insertion and exhibit a 6bp deletion (blue), compared to outgroup species. Note that *Aulonocara baenschi* is heterozygous. Most individuals of *Astatotilapia calliptera* carry the rock- insertion allele; however, individuals at Chizumulu Island and Itupi possess the sand allele.

## Supplementary Figure 5


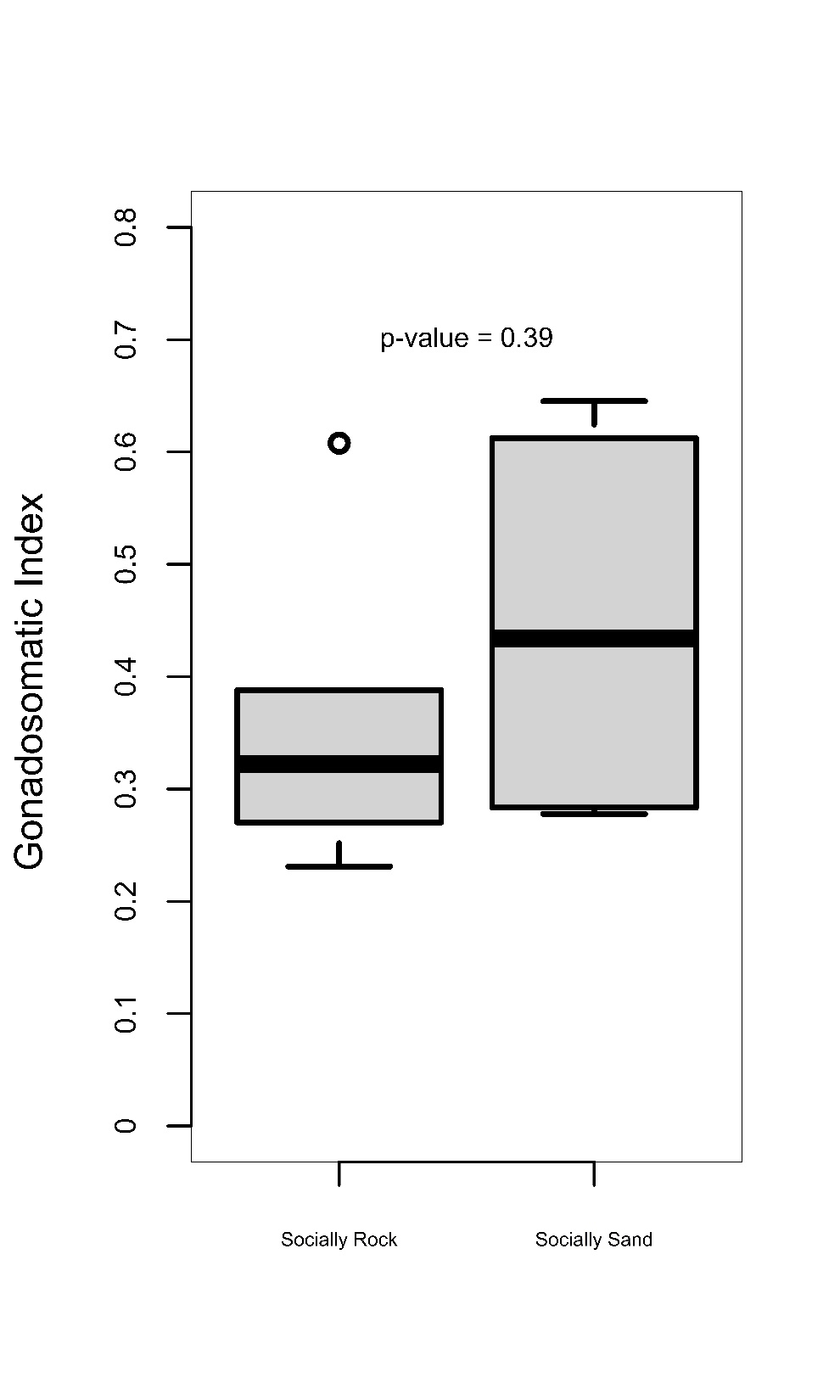


Supplementary Figure 5: Gonadosomatic index (GSI) does not differ amongst males behaving as social rock- or social sand-. See also Figure 3.

## Supplementary Figure 6


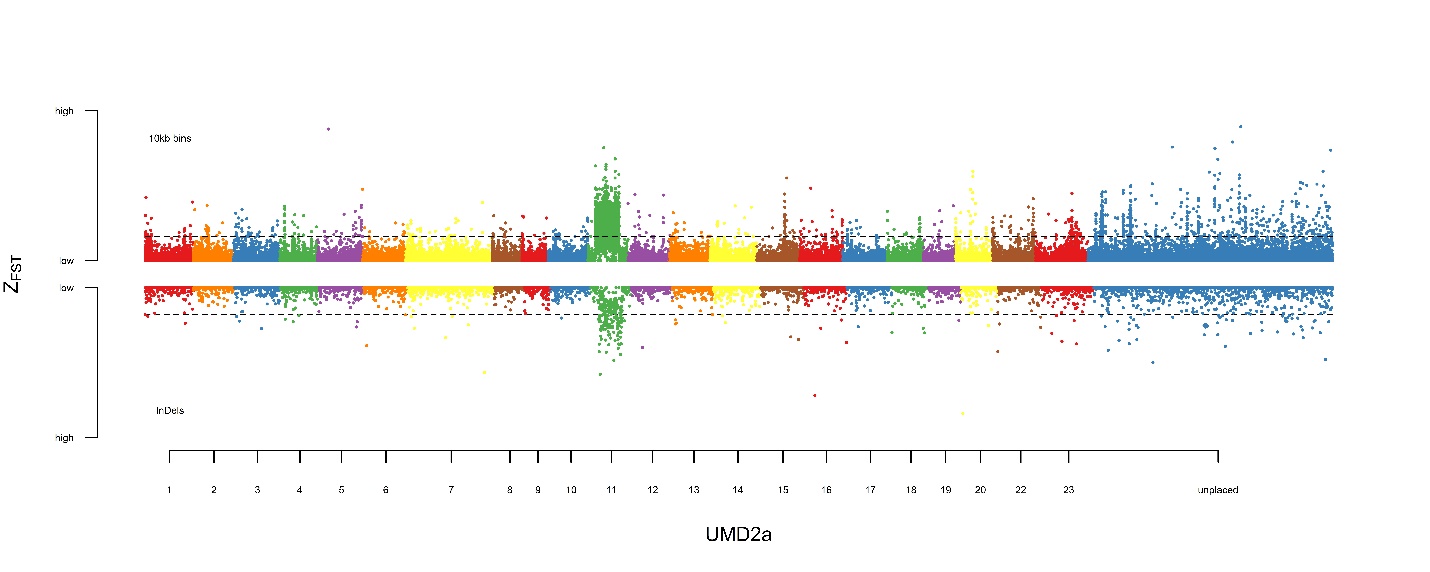


Supplementary Figure 6: Genomic differentiation amongst sand-dwelling species that construct pit vs. castle sand bowers **|** Z-F_ST_ plot (ggplot2 v3.3.3 https://ggplot2.tidyverse.org/) shows genome divergence amongst pit-digging vs. castle-building sand-dweller species, updated by mapping variants to the UMD2a reference genome (data from ^5^). Threshold lines indicate 2.5% FDR.

References

1 Bloomquist, R. F., Fowler, T. E., Sylvester, J. B., Miro, R. J. & Streelman, J. T. A compendium of developmental gene expression in Lake Malawi cichlid fishes. *BMC Dev Biol* **17**, 3, doi:10.1186/s12861-017-0146-0 (2017).

2 Bonkowsky, J. L. *et al.* Domain-specific regulation of foxP2 CNS expression by lef1. *BMC Dev Biol* **8**, 103, doi:10.1186/1471-213X-8-103 (2008).

3 Shi, Z. *et al.* miR-9 and miR-140-5p target FoxP2 and are regulated as a function of the social context of singing behavior in zebra finches. *J Neurosci* **33**, 16510-16521, doi:10.1523/JNEUROSCI.0838-13.2013 (2013).

4 Waterhouse, A. M., Procter, J. B., Martin, D. M., Clamp, M. & Barton, G. J. Jalview Version 2--a multiple sequence alignment editor and analysis workbench. *Bioinformatics* **25**, 1189-1191, doi:10.1093/bioinformatics/btp033 (2009).

5 York, R. A. *et al.* Behavior-dependent cis regulation reveals genes and pathways associated with bower building in cichlid fishes. *Proceedings of the National Academy of Sciences* **115**, E11081-e11090, doi:10.1073/pnas.1810140115 (2018).
