# Supplementary figures and images for: Genome-enabled discovery of evolutionary divergence in brains and behavior
Source: Sci Rep. 2021 Jun 21;11:13016. doi: 10.1038/s41598-021-92385-8 (PMC8217251; doi:10.1038/s41598-021-92385-8)

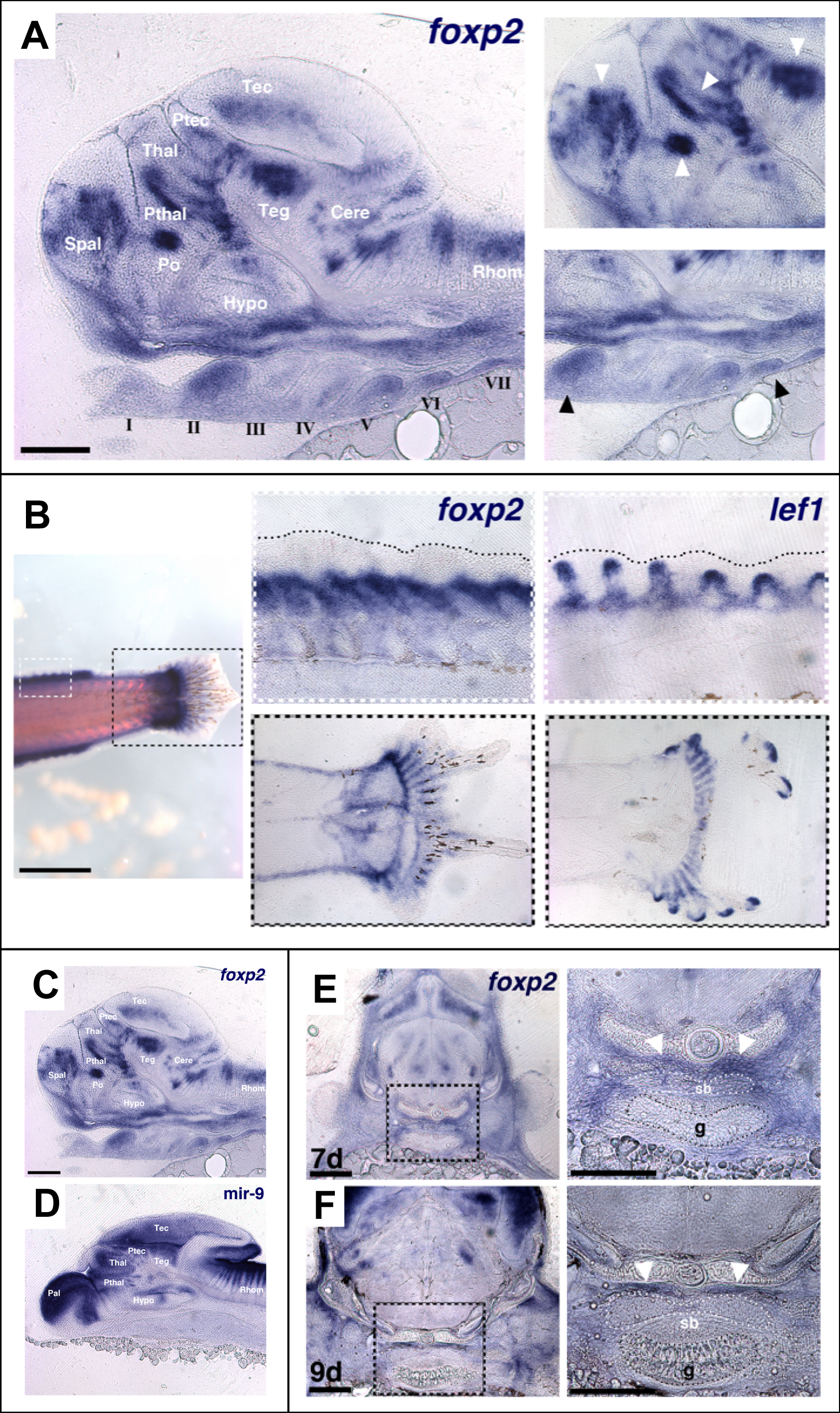

Supplement: Supplementary file 3 — Supplementary Figure S2. [file 41598_2021_92385_MOESM3_ESM.tif]

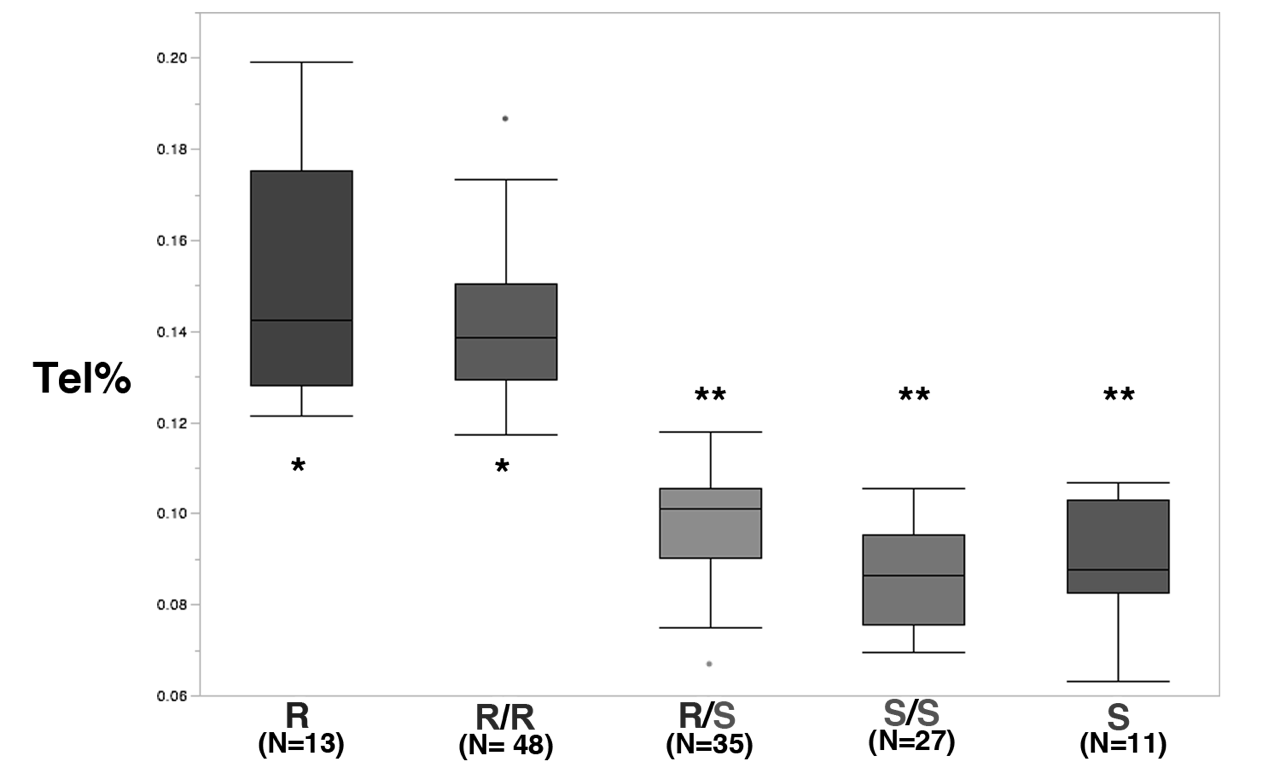

Supplement: Supplementary file 4 — Supplementary Figure S3. [file 41598_2021_92385_MOESM4_ESM.tif]

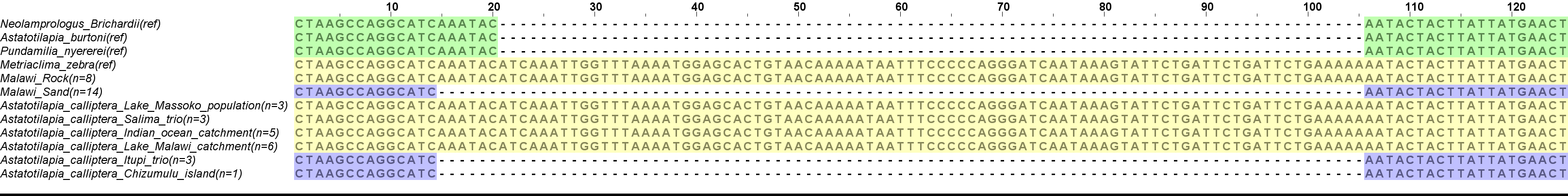

Supplement: Supplementary file 5 — Supplementary Figure S4. [file 41598_2021_92385_MOESM5_ESM.png]
